# Genetic diversity and population structure of *Polistes nimpha* based on DNA microsatellite markers

**DOI:** 10.1007/s00040-015-0421-7

**Published:** 2015-07-07

**Authors:** K. B. Kozyra, I. Melosik, E. Baraniak

**Affiliations:** Department of Systematic Zoology, Adam Mickiewicz University in Poznań, Umultowska Str. 89, 61-614 Poznań, Poland; Department of Genetics, Faculty of Biology, Adam Mickiewicz University in Poznań, Umultowska Str. 89, 61-614 Poznań, Poland

**Keywords:** *Polistes nimpha*, Genetic diversity, Population structure, Microsatellites, Relatedness

## Abstract

The Eurasiatic *Polistes nimpha* belongs to primitively eusocial wasps for which no data are available on its population’s genetic structure and relatedness/relationships of individuals. The purpose of this research is to determine the amplification efficiency in *P. nimpha* of microsatellite primers developed for *P. dominula* and using these primers, to explore genetic diversity, population structure and relatedness/relationship of *P. nimpha* in the context of its reproductive options. Eight out of twelve microsatellite markers analyzed on 59 individuals (pupae and larvae) were polymorphic (mean PIC = 0.545) and mutated following the stepwise mutation model. The Bayesian clustering method gave the probability of >0.898 of there being 10 clusters within the pooled sample of 15 nests. In two or three nest clusters, full- and/or half-siblings and unrelated individuals occurred. A significant correlation between genetic and geographic distances was detected. There are three main possibilities that come into play to explain our genetic results and direct field observations: cooperative nest foundation, visitations, and/or usurpation events. So far there is no conclusive evidence to exclude or support any of these possibilities.

**Electronic supplementary material:**

The online version of this article (doi:10.1007/s00040-015-0421-7) contains supplementary material, which is available to authorized users.

## Introduction

The genus *Polistes* (Hymenoptera: Vespidae) comprises more than 200 wasp species representing a primitively eusocial group of insects (Carpenter 1996). Several factors have led to the intensive study of these species using either traditional (morphologic, behavioral and/or ecologic data) or molecular approaches. These are, for example, interesting population structure and genetics, a wide range of diversity in nesting habits and life cycle, and their role in ecosystems (Pardi 1948; Ono 1989; Sayama and Takahashi 2005; Hughes et al. 2008; Miyano et al. 2010; Nagamati Jr. et al. 2010; Tsuchida et al. 2014).

There are a limited number of studies that have been devoted to identification and the analysis of microsatellite loci in *Polistes* (Strassmann et al. 1997; Henshaw 2000; Saigo and Tsuchida 2010; Henshaw et al. 2011; Uddin and Tsuchida 2011; Komatsu et al. 2012). Here, for the first time, we performed microsatellite analyses of genetic diversity and structure of the population of Eurasiatic *Polistes nimpha* (Christ 1791) (Polistinae).

*Polistes nimpha* is one of the three species of the genus that can be found in Poland (Oleksa and Wiśniowski 2005). Small nests of this species are located on dead stalks of plants predominantly from the Apiaceae and Hypericaceae families, less often on small trees or on blades of grass (up to 50 cm aboveground surface).

The life cycle of *P. nimpha* is similar to that of the other representatives of the genus *Polistes* (see West-Eberhard 1969; Wilson 1979). Spring foundresses of *Polistes* wasps can make different choices regarding reproductive options (e.g., Seppä et al. 2012). Besides haplometrotic nest foundation (nest is founded by a single foundress), pleometrotic nest foundation (nest is started by several queens) is a widespread phenomenon among many species of *Polistes* (Makino and Sayama 1991). The results obtained so far indicate, that in the majority of cases *Polistes* colonies are founded by a single foundress (Miyano and Hasegawa 1998; Sayama and Takahashi 2005), but in *P. nimpha* pleometrotic nest foundation in sheltered places such as buildings was also noted (Cervo and Turillazzi 1985). Moreover, nest usurpation (queen replacement), either intra- or interspecific, in this species was observed (Cervo et al. 2004; Cervo 2006; Lorenzi et al. 2007). Although the other behavioral choices of foundresses during the pre-emergency period were described in *P. dominula* Christ 1791 (Nonacs and Revee 1995) and *P. carolina* (L. 1767) (Seppä et al. 2012), there is no evidence of visiting, deserting, switching, moving, adopting, or nest joining in *P. nimpha*. There is also no evidence of how reproduction is partitioned in the nest between queens and subordinate foundresses and/or potential joiners, movers, or adopters. Whether queens of *P. nimpha* mate with a single or multiple males also has not been tested (but see Turillazzi and Cervo 1982).

This study represents the first attempt to understand genetic diversity and population structure and relatedness/relationship between individuals of *Polistes nimpha.* The purpose of this research is twofold: (1) to determine the amplification efficiency and polymorphism of *P. dominula* microsatellites (Henshaw 2000) in *P. nimpha* and (2) to explore genetic evidence for possible reproductive options in this species.

## Materials and methods

### Study area

**Fig. 1:**
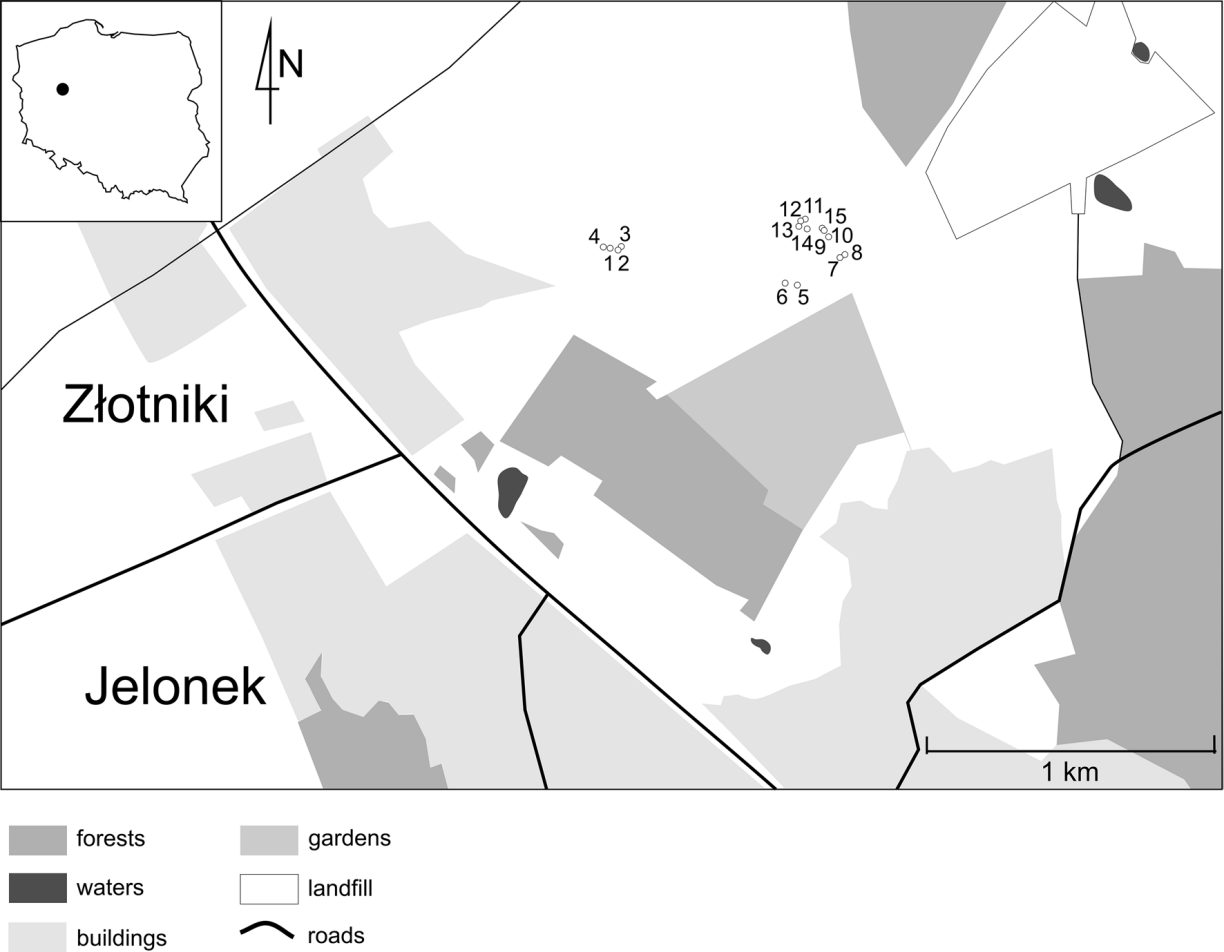
Location of *Polistes nimpha* nests (1–15) in Poland

The study area was located at the edges of a military training field near the city of Poznań, Poland (Fig. 1). Three area plots (17,400; 5300; and 14,200 m^2^) where nest sites occur were selected in April 2014.

### Nest searching and monitoring

Established plots were searched thoroughly for nests of *P. nimpha*. The initial nest searching began on May 12, 2014, and each recorded nest was revisited at approximately 7–10 (21)-day intervals. Nests were watched at forenoon hours 8:00 a.m. to 12:00 noon during the active hours of the inhabitants. One week after the nest identification, from May 19 to May 23, 2014, foundresses of *P. nimpha* were marked individually. Each nest was observed for 45 min per week during the following 5 weeks until days after the first workers’ appearances. Nest contents, a number of cells, and causes of nest failure were documented. Nests were considered as inactive if foundress was not present on the nest for two consecutive following checks. All nests have been marked with numbered tags. Moreover, the nest locations were measured at their latitude and longitude coordinates.

### Sample collection

For genetic analysis, fifty-nine individuals were collected on June 9, 2014 from 15 nests (two pupae and two larvae per nest), except one nest from which one pupa and two larvae were taken. The pupae and larvae were preserved in 95 % alcohol and stored at low temperature (4 °C). Vouchers are deposited in the Department of Systematic Zoology, Faculty of Biology, Adam Mickiewicz University in Poznań, Poland.

### DNA extraction and microsatellite amplification

Genomic DNA was extracted from the legs of each pupa (right leg of the second pair) or from ca. $1/3{-}1/2$ of the larvae body using DNeasy Blood and Tissue Kit (Qiagen GmbH, Hilden, Germany) according to manufacturer’s instructions.

The PCR primers used were those developed for *P. dominula* (Henshaw 2000). PCRs were carried out in 5 μl reaction volumes containing 1× Type-it Microsatellite Kit (Qiagen), 0.07 μM forward primer, 0.2 μM reverse primer, 0.14 μM fluorescent-labeled M13 primer, and 1 μl (6–65 ng) of DNA template using a thermocycling profile of one cycle of 5 min at 96 °C followed by 30 steps of 30 s at 95 °C, 90 s at 50 °C, and 30 s at 72 °C, with a final step of 30 min at 65 °C.

Amplicons were fluorescently labeled in multiplexed reactions using a modified M13-tailing method (Oetting et al. 1995). The amplified alleles were separated on an ABI PRISM 3130XL (Applied Biosystems) with Genescan 600LIZ size standard and scored with the program Peak Scanner v 1.0 (Applied Biosystems).

### Genetic diversity analysis

Genetic analyses were completed on a pooled sample of 59 individuals (pupae and larvae) collected from 15 nests. To describe informativeness of genetic markers related to expected heterozygosity, the polymorphism information content (PIC) values were calculated using the program Cervus. The program Micro-Checker v. 2.2.3 (Van Oosterhout et al. 2006) was used to test for the presence of null alleles, stuttering during the PCR amplification, and large allele dropout.

Genotype frequencies at any locus are a function of allele frequencies in the absence of migration, mutation, assortative mating, and natural selection. This situation is termed Hardy–Weinberg equilibrium. Deviations from this equilibrium can imply inbreeding, physical mixing of populations (Wahlund effect), population stratification, and/or null alleles. The deviation from Hardy–Weinberg proportions caused by heterozygote excess was tested with a global test (*U* test), as implemented in the program GenPop v. 4.2 (Raymond and Rousset 1995; Rousset 2008, http://kimura.univ-montp2.fr/~rousset/Genepop.htm), using 1000 dememorization steps, 100 batches, and 1000 iterations per batch. To assess the Hardy–Weinberg equilibrium for each locus, the goodness-of-fit Chi-square tests (Weir 1996) with Bonferroni correction, as implemented in the program Cervus v. 3.0.6., were performed.

To assign individuals to groups based on their multilocus genotypes, a Bayesian clustering algorithm was used, as implemented in the BAPS software version 6.0 at http://www.helsinki.fi/bsg/software/BAPS (Corander et al. 2008). The data were analyzed using a genetic admixture model for unlinked markers with the a priori upper bound for the number of clusters equal to 15. A principal coordinate analysis (PCoA), available in the program GeneAlEx v. 6.501 (Peakall and Smouse 2006, 2012, http://www.anu.edu.auG/BoZo/enAlEx) was conducted using Nei’s unbiased distance pairwise matrix to test whether patterns in microsatellite data support the partitioning of the sample into BAPS clusters.

As suggested by Frantz et al. (2009), it is important to test the analyzed data for isolation-by-distance before applying the Bayesian method of clustering. The relationship of genetic similarity and geographic distance is expected to be linear under the isolation-by-distance hypothesis (Rousset 1997). To check this hypothesis, a paired Mantel test (Mantel 1967) was performed for analyzed individuals with the program GeneAlEx. The test was based on linear genetic distances and geographic distances calculated on UTM coordinates of Lat./Log.; regression significance was tested with 999 permutations.

Kinship analysis, which relies on a model that assigns dyads to discrete relationship categories (Weir et al. 2006), was performed, based on three estimators: Wang’s (2002), Queller and Goodnight’s (1989) and the maximum-likelihood estimator (Kalinowski and Taper 2006). These estimators are implemented in the programs Coancestry v. 1.0.1.5 (Wang 2011) and ML-Relate (Kalinowski and Taper 2006). A correlation coefficient (*r*) was calculated to measure associations between the relatedness estimators using Statistica v. 10.0 (Statsoft). A *t* test was performed with the same program to determine whether differences between means of relatedness are significant between distinguished groups.

## Results

### Systematic survey of nests

**Fig. 2:**
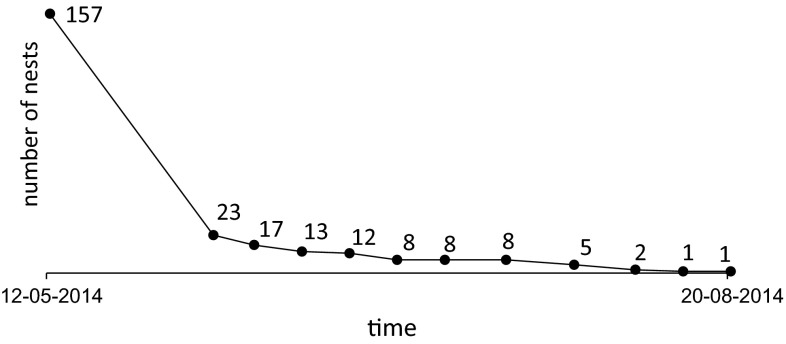
Number of nests of *Polistes nimpha* on the study area (Poland) recorded from May to August 2014

One hundred fifty-seven nests of *Polistes nimpha* were initially found on the selected area plots. A repeat systematic survey of nests has shown that the number of nests recorded decreased substantially by 85.30 % in a short time (the first 3 weeks) (Fig. 2). At the time when pupae and larvae were collected (June 9, 2014), only 17 nests had survived (Fig. 2). Materials for genetic analyses were collected from 15 nests because in the two remaining nests the number of individuals was low. More intense sampling during this phase might diminish substantially or destroy the local population.

The nest destruction was not witnessed, but trace evidence examinations allowed us to conclude that activities of large animals (e.g., foxes), invertebrates (e.g., ants) and/or humans had destroyed the nests. In over 80 % of the cases, the mammals’ activity was the most important predictor of nest mortality. For a large proportion of nests, the reason for their failure is unknown. The degree of destruction varied substantially across sites; only stalks of nests remained (the comb had been damaged), or inhabitants had been eaten, but the nest (comb) had remained untouched (Table 1).

**Table 1:** Percentage of destroyed and abandoned nests of *Polistes nimpha* and external sources of nests destruction based on observations made at three plots in the Wielkopolska region (Poland) in 2014

Causes of mortality	Date of observation											
	May 12	Jun 5	Jun 11	Jun 18	Jun 25	Jul 2	Jul 9	Jul 18	Jul 28	Aug 6	Aug 13	Aug 20
Mammals’ activity	0	70.70	0.64	0	0	0	0	0	1.91	0	0.64	0
Ants’ activity	0	1.91	0	0	0	0	0	0	0	0	0	0
Anthropogenic activity	0	0	0	0	0	0.64	0	0	0	0	0	0
Abandoned nests	0	1.91	0	0	0	0	0	0	0	0.64	0	0
Undetected causes of nest failure	0	10.83	3.18	2.55	0.64	1.91	0	0	0	0	0	0
Total % of nests												
Active	100	14.65	10.83	8.28	7.64	5.09	5.09	5.09	3.18	1.27	0.64	0.64
Destroyed	0	83.44	3.82	2.55	0.64	2.55	0	0	1.91	1.91	0.64	0

Due to the considerable nest mortality (Fig. 2), only 42 foundresses (of 157 initially identified nests) were marked. Eleven marked foundresses originated from nests sampled for genetic analyses. Based on the mobility behavior of marked foundresses, we can conclude that all these individuals have been observed only on their natal nests. However, not marked, visiting, or usurping (not aggressively behaved) wasps were observed during weekly inspections of localized colonies of *P. nimpha* on May 8, 2014 and May 14, 2014.

### Microsatellite cross-species amplification

High rates of microsatellite amplification were observed. From a total of 13 primer pairs, 12 pairs that amplify without any problems were chosen for genetic analysis. Table S1 shows the characteristics of the pursued and nonamplifiable pair of primers. Eleven microsatellite loci (*Pdom 1, 2, 7, 20, 25, 93, 117, 121, 127, 139, 140*) were polymorphic, and one locus (*Pdom 151*) was monomorphic (Table S1). Only eight polymorphic loci had a size variation consistent with the occurrence of strictly stepwise mutations in the repeat array. The remaining primers (*Pdom 25, 117, 121*) showed more complex patterns of evolution (nonstepwise mutations). These three loci were excluded; thus the truncated set contains data for eight microsatellite loci collected from 59 individuals. Large allele dropout and stuttering were not detected. At the locus *Pdom 7*, missing data for four individuals probably appeared because of mutations in the primer binding sites.

### Genetic diversity and structure within the pooled sample

Standard measures of genetic diversity in *P. nimpha* population were calculated. The mean PIC score of the microsatellite loci (*n* = 8) was equal to 0.545, ranging from 0.033 (*Pdom 93*) to above 0.5 (*Pdom 1, 139, 7, 127*). The latter four loci are highly polymorphic. The mean number of different alleles per locus was 6.875, ranging from 2 (*Pdom 93*) to 11 (*Pdom 7*). The mean expected heterozygosity was 0.5765 (unbiased expected heterozygosity UHe = 0.577), meaning that there is a >57 % chance of being a heterozygote under random mating conditions. The average value of the observed heterozygosity (*H*_o_) was slightly higher than Hardy–Weinberg expectations, *H*_o_ = 0.588. However, no significant deviations from Hardy–Weinberg equilibrium were observed for the alternative hypothesis of heterozygote excess (H1), (*P* = 0.433 ± SE 0.020). Chi-square tests for individual loci showed a significant deviation (*P* < 0.05) for *Pdom 20* (Chi-square test value using Yates’s correction = 7.622), (Table S2).

**Fig. 3:**
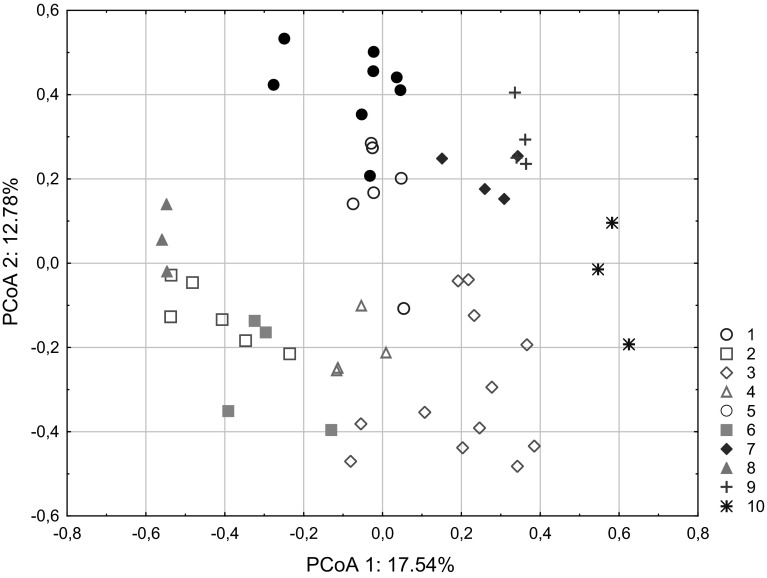
Principal coordinate plot of *Polistes nimpha* individuals based on Nei’s standard distances. Axis 1 explains 17.54 % of the variation, and axis 2 explains 12.78 % of the variation. Clusters of individuals (clusters 1–10 marked with *different colors*) were obtained in a BAPS analysis (Bayesian analysis of population structure) (color figure online)

**Fig. 4:**
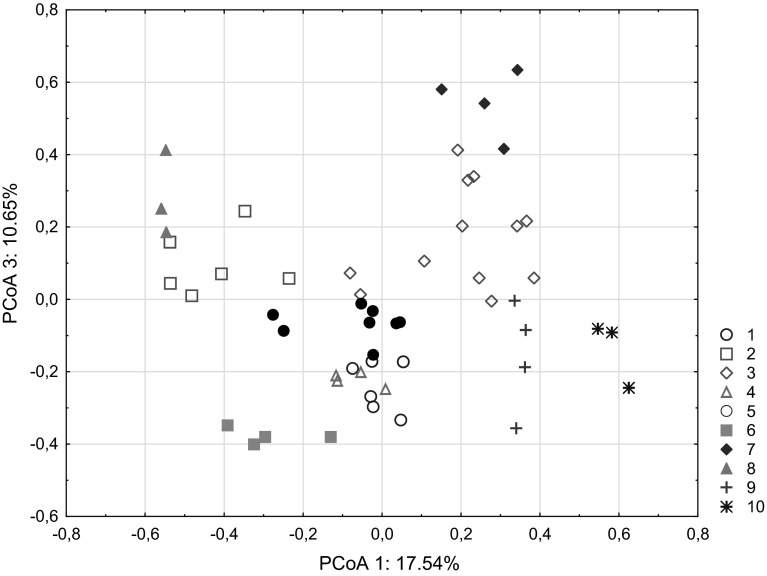
Principal coordinate plot of *Polistes nimpha* individuals based on Nei’s standard distances. Axis 1 explains 17.54 % of the variation, and axis 3 explains 10.65 % of the variation. Clusters of individuals (clusters 1–10 marked with *different* *colors*) were obtained in a BAPS analysis (Bayesian analysis of population structure) (color figure online)

To relate observable structure (nests) to the genetic structure of analyzed population, a model-based clustering was employed. Taking genetic information into account, the Bayesian clustering method, based on the individual-level clustering model for unlinked markers, gave the probability of >0.898 of being 10 genetic clusters within the pooled sample of 15 nests (log marginal likelihood of optimal partition = –1025.9824). The inferred six clusters (4, 6–10) were coherent in terms of wasps’ nests (i.e., each cluster contains individuals originated from one nest). The remaining clusters (1–3, 5) were composed of all individuals originated from two nests (clusters 1, 2, and 5) or three nests (cluster 3). The principal coordinate analysis (PCoA) results broadly confirmed the grouping based on BAPS analysis. In the PCoA, the individuals were grouped accordingly, but only 40.97 % of the variation in the input data has been captured by the first three axes (PCoA 1–3), (Figs. 3 and 4). All individuals studied belong to the same generation and based on genetic data all were heterozygous at least at one of analyzed loci. Thus, they represent presumably the same sex (worker-like females), (see Balloux and Lugon-Moulin 2002, Lorenzi 2009).

In the case of organism’s limited dispersal ability, we expect an isolation-by-distance type of population structure, in which genetic similarity is contrarily related to geographical distance calculations (Meirmans and Van Tienderen, 2012). The comparison of linear genetic distances with geographic distances by the Mantel test revealed a significant correlation between genetic and geographic distances (*R*^2^ = 0.0413, *P* < 0.001); thus, it can be concluded that genetic differences among individuals do increase linearly with geographic distance.

**Fig. 5:**
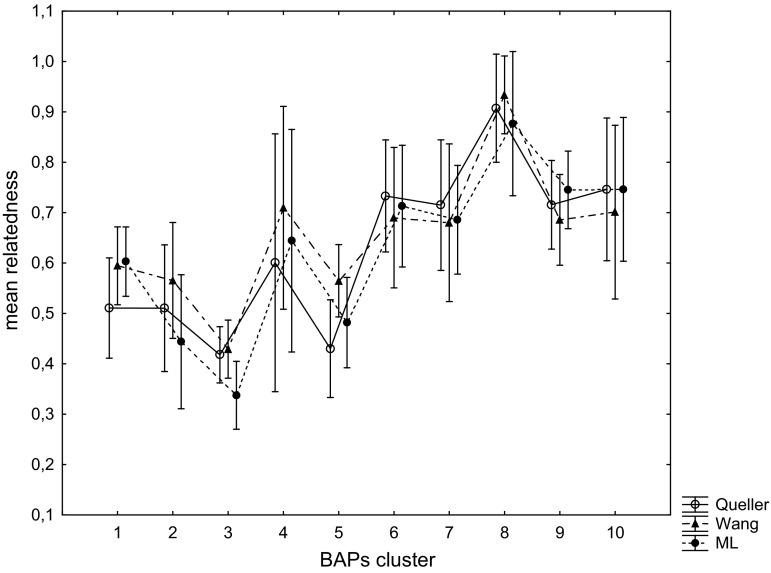
Mean and 95 % confidence interval for relatedness coefficients were based on the method-of-moments approach (Queller and Goodnight 1989; Wang 2002) and maximum-likelihood approach (ML estimator) (Kalinowski and Taper 2006) between all possible dyads of *Polistes nimpha* within clusters of individuals obtained in a BAPS analysis (Bayesian analysis of population structure)

**Fig. 6:**
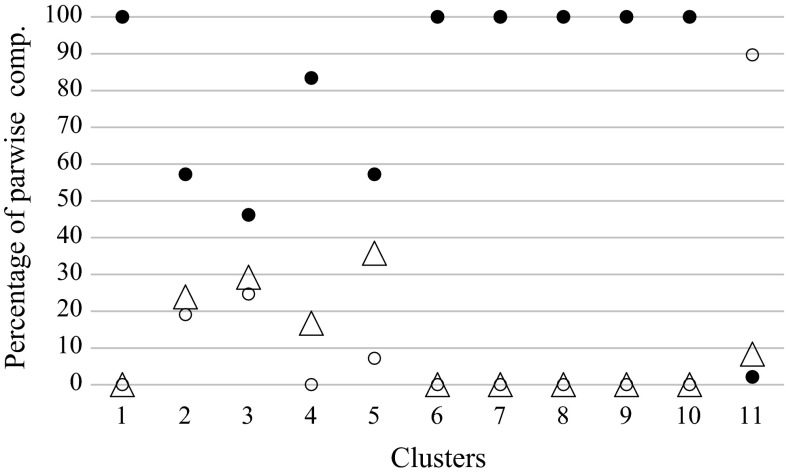
Percentage of pairwise comparisons between individuals of *Polistes nimpha* divided into relationship categories (full-sibs, half-sibs and unrelated) based on the maximum-likelihood estimator of Kalinowski and Taper (2006) within clusters of individuals obtained in a BAPS analysis (Bayesian analysis of population structure), and between clusters (these denoted as “11”). Legend: full-siblings (*black circles*), half-siblings (*triangles*), and unrelated individuals (*white circles*)

The three metrics of relatedness employed were highly correlated (*n* = 1711, all *r* > 0.91). Based on the method-of-moments estimators, the BAPS clusters composed of individuals originated from two or three nests (considered as a group 1, nos. 1–3, 5) expressed significantly lower levels of relatedness coefficients (*P* < 0.05) than did the second group composed of one-nest clusters (nos. 4, 6–10) (Table S3; Figs. 5 and 6). The ML estimator did not show significant differences between these groups.

Discriminations among plausible relationships (full-sibs, half-sibs, and unrelated) were based on the maximal likelihood. All pairwise comparisons between individuals classified to mixed cluster no. 1 (composed of two nests, nos. 2 and 9, Fig. 1) were classified as full-siblings or parental offspring (here, both categories were considered jointly as full-siblings). However, the mean value of relatedness within this cluster was significantly lower than that within the one-nest clusters (Table S4). In the mixed clusters (nos. 2–3 and 5), the percentage of pairwise comparisons classified as full-siblings ranged from 46.15 to 57.14 %; as half-siblings, from 23.81 to 35.71 %; and as unrelated individuals, from 7.14 to 24.615 % (Fig. 6). In one-nest clusters (nos. 6–10), only full-siblings were found. In one-nest cluster no. 4, the majority of pairwise comparisons were classified as full-sibs, whereas one-sixth were classified as half-siblings; no unrelated individuals were found.

## Discussion

Eight loci out of 12 analyzed can be effectively used to genotype individuals of *P. nimpha.* These most variable perfect loci have the average expected and observed heterozygosities over 0.57, that are comparable but slightly lower than those detected for *P. dominula* (Henshaw 2000; Henshaw et al. 2011), *P. chinensis antennalis* (Saigo and Tsuchida 2010), and *P. satan* (Komatsu et al. 2012).

In the cases of limited number of microsatellite loci (5–20), the method-of-moments estimators is commonly preferred (Csilléry et al. 2006). Nevertheless, different relatedness estimators employed here yielded highly correlated estimates of relatedness between pairs of *P. nimpha* individuals. Therefore, besides the commonly used, the Queller and Goodnight’s (1989) estimator that was developed for haploid–diploid social Hymenoptera, the maximum-likelihood (ML) estimators (Milligan 2003, Kalinowski and Taper 2006) can be successfully employed in the further studies of social insects.

The Bayesian analysis provided support for the existence of genetic structure in the analyzed population of *P. nimpha* by partitioning individuals into 10 clusters. This grouping was also broadly confirmed by principal coordinate analyses. The existence of mixed BAPS clusters (i.e., composed of two or three nests) in which only some individuals that originated from different nests are full-sibs and the others are unrelated may indicate three main reproductive tactics in the population of this species: short visits of foundresses, cooperative (pleometrotic) nest foundations, and/or nest usurpation. These tactics in *Polistes* wasps are hypothesized to have different fitness payoffs over time, depending on environmental shifts (Starks and Fefferman 2006; Seppä et al. 2012).

Pleometrotic nest foundation was described for *Polistes* species, e.g., European *P. dominula* (Pardi 1942; Nonacs and Reeve 1995), American *P.* c*arolina* (L.) (Seppä et al. 2012), neotropical *P. versicolor* (Olivier) (De Oliveira et al. 2010 and references therein), and *P. nimpha* (Turillazzi and Cervo 1985). As was hypothesized by Gamboa (1980) and Tibbetts and Reeve (2003), multiple foundresses increase colonies’ productivity and survivorship and make them less likely to be usurped. Nevertheless, this tactic may negatively affect the nest if foundresses are infected by parasites (Hodges et al. 2003 and references therein). To some extent, pleometrotic nest foundation is contradicted by our evidence of marked foundresses of *P. nimpha* that were seen only on natal (single) nests. Nevertheless, this kind of cooperation cannot be fully excluded due to methodological reasons (marking of wasps was performed shortly after eggs were laid but before offspring emerged). So far, in *P. nimpha*, pleometrotic nest foundation was detected only at sites where the aggregation of sibling females was possible during the winter (i.e., “in covered and sheltered” places, such as buildings) (Turillazzi and Cervo 1985: 49). The winter destruction of plants and difficulties in the aggregation of females that build nests on this kind of substratum were hypothesized to be main factors for nest foundation by a single foundress (Turillazzi and Cervo 1985). Based on materials, we collected from the nests built on vegetation; the single-foundress colonies comprise only 50 % of detected BAPS clusters. Thus, the causes of pleometrotic nest foundation have yet to be investigated and determined.

Nevertheless, other possibilities also should be considered to interpret population structure of *P. nimpha* from our genetic data. The possibilities of laying eggs by visiting, nonaggressive wasps in the nests of sibling females or intraspecific usurpation cannot be excluded based on our direct field observations. It should be underlined, however, that in the case of unmarked wasps, there are always difficulties in differentiating a visitor, a joiner, a switcher, an adopter, or a usurper (Nonacs and Reeve 1985). The case of cluster no. 1 being composed of two nests in which full-siblings were found is a prime example of ambiguity because the interpretation could be twofold: eggs were laid by one queen who was fertilized by related males, or eggs were laid by a pair of full sisters. The former phenomenon was discovered for the first time by Seppä et al. (2011) in *P. biglumis* L.

The results of the comparison of the genetic distances to the geographic distances with the Mantel test suggest that individuals from a particular nest are more genetically related to those inhabiting nearby nests than they are to individuals originating from nests situated in further parts of the study area. Foundresses of the studied nests may be the offspring of one or several closely related queens that returned to their natal sites after hibernation. Cervo and Turillazzi (1985) observed that new colonies of *P. nimpha* in synanthropic conditions were initiated very closely to the natal nest. It has been shown that the recognition of the natal nest is possible due to the hydrocarbon profile specifics of each *Polistes* colony (Gamboa 2004; Sumana et al. 2005). Philopatric tendencies in some species of *Polistes* wasps were observed previously by West-Eberhard (1969), Klahn (1979), Hirose and Yamasaki (1984), Makino et al. (1987) and Cervo and Turillazzi (1985). For example, most colonies of Japanese *Polistes* wasps are initiated within a distance of 50–80 m (164.5–263 feet) from the natal nest (Hirose and Yamasaki 1984; Makino et al. 1987). Nevertheless, no isolation by distance among populations of *P. olivaceus* (De Geer) was detected (Uddin and Tsuchida 2011).

However, the tendency of individuals originating from two or three different nests to cluster together can reflect close affinity of some individuals or be a result of stochastic error (Kalinowski 2011). The number of clusters detected by Bayesian clustering algorithms does not always correspond to the number of biologically meaningful populations (groups) in the studied sample (Francois and Durand 2010). Inference of population structure is sensitive to the number of loci scored, the number of populations, the number of individuals per population, the sampling strategy, and the chosen analysis method (e.g., Evanno et al. 2005; Rodriguez-Ramilo et al. 2009; Francois and Durand 2010; Kalinowski 2011; Putman and Carbone 2014). Moreover, as was shown by Frantz et al. (2009), the Bayesian methods employed in BAPS overestimate genetic structure when one or more loci significantly deviate from the Hardy–Weinberg equilibrium and when isolation by distance exhibits high levels (*b* ≤ −0.01).

Similar to the majority of wildlife studies, this study suffers from several limitations resulting from the small number of available loci for analysis, the relatively small sample size, and the small size of the area from which studied individuals were collected. Moreover, based on data sets analyzed here, isolation-by-distance and a significant deviation from Hardy–Weinberg proportions for one locus were detected. Thus, the presented results should be treated with caution until more data are collected.

A large number of colonies established by paper wasps are relatively soon completely destroyed or substantially damaged by natural enemies such as predators and parasites and by human activity of various kinds (Strassmann 1981; Makino 1983; Cervo and Turillazzi 1985; Rusina 2011) The highest rates of nest mortality, attributable to an external agent, were observed in *P. nimpha* during the first weeks after nest identification. After nest destruction, foundresses can choose the following options: nesting alone (nest rebuilding), usurping established colonies, or joining other queens (Nonacs and Reeve 1985). Based on our data, no nests rebuilding or joining has been observed (see also Makino 1989). Therefore, dispersing or dying of foundresses has to be considered to explain this observation.

In conclusion, our study demonstrates for the first time a useful panel of microsatellite markers to perform population genetics studies dealing with *P. nimpha.* There are three main possibilities that come into play to explain our genetic results and direct field observations: cooperative nest foundation, visitation, and/or usurpation events. So far, there is no conclusive evidence to exclude or support any of these possibilities. A philopatric behavior of foundresses is suggested based on genetic data. However, further and more extensive studies in terms of the number of individuals and loci are needed.

## Electronic supplementary material

Supplementary material 1 (PDF 122 kb)

Supplementary material 2 (PDF 195 kb)

Supplementary material 3 (PDF 111 kb)

Supplementary material 4 (PDF 191 kb)
