# Supplementary material for: Genetic diversity and population structure of Polistes nimpha based on DNA microsatellite markers
Source: Insectes Soc. 2015 Jul 7;62:423–32. doi: 10.1007/s00040-015-0421-7 (PMC4768218; doi:10.1007/s00040-015-0421-7)
Supplement: Supplementary file 1 — Supplementary material 1 (PDF 122 kb) [file 40_2015_421_MOESM1_ESM.pdf]

Genetic diversity and population structure of *Polistes nimpha* (Hymenoptera: Vespidae) based on DNA microsatellite markers

Insectes Sociaux

Krzysztof Kozyra, Iwona Melosik, Edward Baraniak

Corresponding author: Iwona Melosik, Department of Genetics, Faculty of Biology, Adam Mickiewicz University in Poznań, Umultowska Str. 89, 61-614 Poznań, Poland.

melosik1@amu.edu.pl, phone (+048) 61 829 58 60

Table S1

Locus performance in the study of *Polistes nimpha* (primers designed by Henshaw 2000 for *P. dominulus*).

Abbreviations: *for primers not used because of poor PCR amplification; **for primers for microsatellites that revealed a complex pattern of mutations (excluded from the genetic analysis)

Locus	Primer sequences	Motive	Dye	Observed ranges (bp)
Pdom20	F:TTC TCT GGC GAG CTG CAC TC<br>R:AGA TGG CAT CGT TTG AAA GAG C	(CAT)18	M13 A VIC	242-254
*Pdom25***	F:CAT TAT AAA CGC CGC G<br>R: ACG ATG GAA ACG TAA GTC C	(AAG)11	M13 G NED	166-202
Pdom93	F:CCA TCA GCT GTC CCA TTC GC<br>R: AAT CGG TTT CGC TCG TCC ACC TCC	$(AAG)_2ACG(AAG)_2ACG(AAG)_5$	M13 C 6FAM	142-145
*Pdom117***	F: AAG AAA ACC TACTAC GTT GTG TGA G<br>R: TTT CAA CAT TCC ATA GGG ACA G	$(AAG)_4AGG(AAG)_2AGG(AAG)_{14}$	M13 T PET	261-292

*Pdom121***	F: GAG TGG GTA TGA CGA AGA TGA TGG<br>R: TGA TTA TAG CCT GCC GAA ACT CTG	$(AAG)_8$AGGAAC$(AAG)_2$AAC$(AAG)_2$	M13<br>A VIC	223-256
Pdom127	F: TCC CCC GTT TTT GGT CCT TG<br>R: GGG AGA GAA TCG TGC CTT TTC	$(AAT)_{13}$...$(AAT)_6$AA$(AAT)_4$AAC(AAT)	M13<br>C<br>6FAM	109-135
Pdom139	F: TGA CAA AAG ACA ACA AAA TAT G<br>R: AGC TTC GGT AGG GCT TCG	$(AAC)_7(AAT)_2(AAC)(AAT)_2(AAC)_2$	M13<br>T PET	206-232
Pdom140	F: GCT TTT CCC TTA TTT TCC CG<br>R: CGT GTT CGT ATA TTC CTG TAA CG	$(TAG)_9$	M13<br>A VIC	218-260
*Pdom151**	F: TGA TGT TAC CAC TGC TTT GAG CG<br>R: TTC AGC ACC GTC GTC GTT GTT G	$(CAT)_2$AA(CAT)CAAT$(CAT)_3$	M13<br>G NED	131
Pdom1	F: GGA CGC TCG GCT GAT TTG TC<br>R: AAG GGA TTT TTC CTG AGA CTA TTC G	(CAG)9TAG(CAG)5(CAT)5<br>GGCAC(CAG)3	M13<br>C<br>6FAM	176-218
Pdom2	F: CGT CTC TCG AAA TAT GCT AAA C<br>R: AGA ACG GTA AAC ATT CTT CTA TC	(AAG)8CG(AAG)2	M13<br>T PET	190-202
Pdom7	F: CAC TGT ATT GTC CTA CGG TGG TCC<br>R: CG AGA ACC TGT ACT CAA AAC AAA C	(AAG)CAG(AAG)9	M13<br>G NED	166-197
