# Supplementary material for: Genetic diversity and population structure of Polistes nimpha based on DNA microsatellite markers
Source: Insectes Soc. 2015 Jul 7;62:423–32. doi: 10.1007/s00040-015-0421-7 (PMC4768218; doi:10.1007/s00040-015-0421-7)
Supplement: Supplementary file 2 — Supplementary material 2 (PDF 195 kb) [file 40_2015_421_MOESM2_ESM.pdf]

Genetic diversity and population structure of *Polistes nimpha* (Hymenoptera: Vespidae) based on DNA microsatellite markers

Insectes Sociaux

Krzysztof Kozyra, Iwona Melosik, Edward Baraniak

Corresponding author: Iwona Melosik, Department of Genetics, Faculty of Biology, Adam Mickiewicz University in Poznań, Umultowska Str. 89, 61-614 Poznań, Poland.
melosik1@amu.edu.pl, phone (+048) 61 829 58 60

Table S2

Summary statistics for eight microsatellite loci analyzed in wasp *Polistes nimpha*; # - number of alleles, *Ho* – observed heterozygosity, *He* – expected heterozygosity, PIC- polymorphism information content, HWE – Hardy-Weinberg Equilibrium (*statistically significant at $P < 0.05$; NS: not significant, ND: not determined), F(null) – frequency of null alleles (negative values arise probably due to a skewed frequency distribution).

Locus	# of alleles	*Ho*	*He*	PIC	HWE	F(null)
Pdom20	5	0.627	0.494	0.443	*	-0.1486
Pdom93	2	0.034	0.034	0.033	ND	-0.0025
Pdom127	9	0.712	0.857	0.832	ND	0.0881
Pdom139	9	0.712	0.737	0.700	NS	0.0124
Pdom140	6	0.576	0.504	0.462	NS	-0.0741

Pdom1	8	0.729	0.712	0.667	NS	-0.0070
Pdom2	5	0.458	0.450	0.423	ND	-0.0132
Pdom7	11	0.855	0.824	0.799	NS	-0.0240
