# Supplementary material for: Genetic diversity and population structure of Polistes nimpha based on DNA microsatellite markers
Source: Insectes Soc. 2015 Jul 7;62:423–32. doi: 10.1007/s00040-015-0421-7 (PMC4768218; doi:10.1007/s00040-015-0421-7)
Supplement: Supplementary file 3 — Supplementary material 3 (PDF 111 kb) [file 40_2015_421_MOESM3_ESM.pdf]

Genetic diversity and population structure of *Polistes nimpha* (Hymenoptera: Vespidae) based on DNA microsatellite markers

Insectes Sociaux

Krzysztof Kozyra, Iwona Melosik, Edward Baraniak

Corresponding author: Iwona Melosik, Department of Genetics, Faculty of Biology, Adam Mickiewicz University in Poznań, Umultowska Str. 89, 61-614 Poznań, Poland.

melosik1@amu.edu.pl, phone (+048) 61 829 58 60

Table S3

Results of *t* test for comparison of relatedness coefficients (Wang 2002, Queller and Goodnight (1989), and Kalinowski (2006) between one-nest clusters and mixed clusters of wasp *Polistes nimpha*; statistical significance at $P < 0.05$.

Relatedness estimator	Mixed clusters Mean ±SD	One-nest clusters Mean ±SD	*t* test	df
Wang (2002)	0.508 ±0.231	0.735±0.159	-5.478*	175
Queller and Goodnight (1989)	0.452±0.245	0.737±0.161	-6.514*	175
Kalinowski (2006)	0.433±0.270	0.733±0.145	-6.413	177
