# Supplementary material for: Genetic diversity and population structure of Polistes nimpha based on DNA microsatellite markers
Source: Insectes Soc. 2015 Jul 7;62:423–32. doi: 10.1007/s00040-015-0421-7 (PMC4768218; doi:10.1007/s00040-015-0421-7)
Supplement: Supplementary file 4 — Supplementary material 4 (PDF 191 kb) [file 40_2015_421_MOESM4_ESM.pdf]

**Genetic diversity and population structure of *Polistes nimpha* (Hymenoptera: Vespidae) based on DNA microsatellite markers**

Insectes Sociaux

Krzysztof Kozyra, Iwona Melosik, Edward Baraniak

Corresponding author: Iwona Melosik, Department of Genetics, Faculty of Biology, Adam Mickiewicz University in Poznań, Umultowska Str. 89, 61-614 Poznań, Poland.

melosik1@amu.edu.pl, phone (+048) 61 829 58 60

Table S4

Descriptive statistics (mean ± SD) of relatedness estimators based on method-of-moments approach (Queller and Goodnight 1989; Wang 2002) and maximum-likelihood (ML) estimator (Kalinowski et al. 2006) calculated within 10 clusters of individuals of *Polistes nimpha* obtained in a BAPS analysis (Bayesian Analysis of Population Structure).

| Relatedness estimator | Wang, 2002 | Queller and Goodnight, 1989 | ML |
|---|---|---|---|
| BAPS cluster | | Mean±SD | |
| 1 | 0.595±0.199 | 0.511±0.257 | 0.603±0.1775 |
| 2 | 0.565±0.253 | 0.511±0.276 | 0.444±0.292 |
| 3 | 0.429±0.233 | 0.418±0,225 | 0.337±0.274 |
| 4 | 0.710±0.192 | 0.6005±0.244 | 0.644±0.210 |
| 5 | 0.565±0.185 | 0.430±0.250 | 0.482±0.231 |
| 6 | 0.690±0.133 | 0.733±0.106 | 0.713±0.115 |

| | | | |
|---|---|---|---|
| 7 | 0.680±0.149 | 0.715±0.123 | 0.686±0.103 |
| 8 | 0.934±0.074 | 0.907±0.102 | 0.877±0.136 |
| 9 | 0.686±0.0725 | 0.716±0.071 | 0.745±0.062 |
| 10 | 0.710±0.164 | 0.746±0.135 | 0.746±0.136 |
